# Competition model explains trends of long‐term fertilization in plant communities

**DOI:** 10.1002/ece3.9832

**Published:** 2023-02-14

**Authors:** Atsushi Yamauchi, Koichi Ito, Shota Shibasaki

**Affiliations:** ^1^ Center for Ecological Research Kyoto University Otsu Japan; ^2^ International Institute for Zoonosis Control Hokkaido University Sapporo Japan; ^3^ Department of Fundamental Microbiology University of Lausanne Lausanne Switzerland

**Keywords:** community structure, competition–fecundity trade‐off, nutrient, rank abundance diagram

## Abstract

Over 40 years ago, Kempton (*Biometrics*, **35**, 1979, 307) reported significant modification to plant community structure following a long‐term fertilization experiment. Many researchers have investigated this phenomenon in the years since. Collectively, these studies have shown consistent shifts in rank abundance relationships among species in communities following fertilization. The previous studies indicated that fertilization affects community structure through several critical processes, including trait‐based functional response, reordering of species in rank abundance diagram (RAD), and niche dimensionality, although some questions have remained. How does the species reordering driven by the plant responses cause characteristic trends in temporal changes of RAD? Why are those trends ubiquitous in various systems? To answer those questions, we theoretically investigated the effects of fertilization on community structure based on a colonization model (or Levins model) with competition–fecundity trade‐offs, which can result in the coexistence of multiple species under competition. The model represents characteristic RAD, which can be an adequate tool to study community composition. Our theoretical model comprehensively represents observed trends in rank abundance relationships following long‐term fertilization and suggests that competitive interactions among species are a critical factor in structuring species diversity in plant communities.

## Graphical abstract

Colonization model explained trends in structural change of plant community that results from long‐term fertilization. Our analyses provide new insight into the formation of community structure and species diversity.

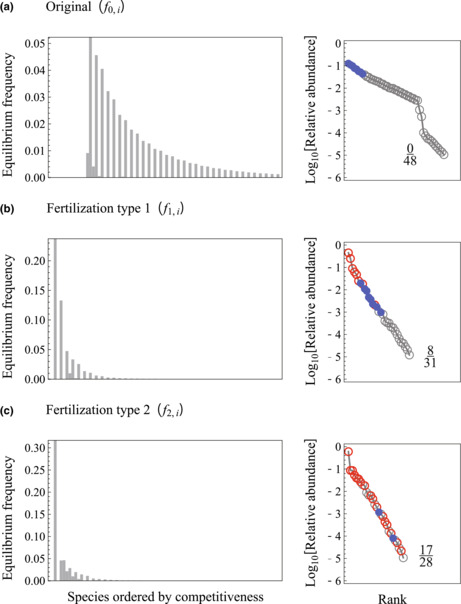

## 1 INTRODUCTION

Species abundance distributions (SADs) characterize the properties of species diversity within communities, and ecologists have long aimed at extracting ecological insight from SADs (McGill et al., 2007; Yamauchi, Tokita, et al., 2021). The shape and nature of a given SAD vary with region, taxonomy, and community history (Magurran, 2004). In addition, SADs can be modified by treatments such as long‐term fertilization (Avolio et al., 2019; Collins et al., 2008; Kempton, 1979; Kirkham et al., 1996; Sand‐Jensen et al., 2008). Changes in community structure following fertilization tend to follow three major trends: species richness declines (Avolio et al., 2019; Collins et al., 2008; Dickson & Gross, 2013; Kempton, 1979; Kirkham et al., 1996; Sand‐Jensen et al., 2008), a situation where the most abundant species are replaced by species that were rare or absent before fertilization (Avolio et al., 2019; Collins et al., 2008; Kirkham et al., 1996; Sand‐Jensen et al., 2008), and a steeping of the slope of the rank abundance diagram (RAD; Collins et al., 2008; Kempton, 1979; Kirkham et al., 1996; Sand‐Jensen et al., 2008). It should be noted that the change in steepness of RAD represents an alternation of species evenness. Community structure changes following fertilization may provide substantial insight into the mechanisms driving species diversity.

Nutrient fertilizations modify community composition through functional responses in the community, including changes in plant trait expressions (La Pierre & Smith, 2015) and changes in abundance of functional groups (Dickson & Gross, 2013; Suding et al., 2005). Brown and Zinnert (2021) recently investigated the effects of fertilization of multiple nutrients using a trait‐based approach, reporting that the trait‐based functional alpha‐diversity positively correlated with the total biomass. Avolio et al. (2019) showed that species reordering in RAD explains community change observed in the long‐term fertilization experiments. Brown et al. (2022) also studied species reordering in a coastal grassland community under multiyear nutrient enrichment, associated with changes in both plant traits and RADs. These studies suggest that fertilization of nutrient(s) causes responses of plants in trait expression, which drives reordering of species abundance, resulting in changes in community structure. Eskelinen et al. (2022) showed that the presence of herbivores and competition for light were critical factors in the decline of plant diversity in nutrient enrichment, which suggested the importance of competition in the response of diversity to nutrient conditions.

On the contrary, some studies clarified a link between interspecific competition for multiple resources and species diversity. Harpole and Tilman (2007) showed simultaneous enrichments of multiple types of nutrients generally decline species diversity. Harpole et al. (2016) subsequently indicated that such a trend did not necessarily associate with biomass increment of the community in some cases. This suggests that resource conditions influence the community structure, whereas biomass is not an essential factor. Accordingly, they concluded that a release from the limitation of multiple nutrients would be a crucial factor for the decreasing diversity, which is consistent with a prediction of niche dimensionality, that is, more limiting factors allow for more ways that species can coexist. This study successfully shows the effect of resource limitation on species diversity, although a question arises in the influence of fertilization. Why did not the improvement of nutrient conditions increase biomass in some systems? Thus, a relationship between fertilization and biomass change should be studied more deeply concerning the change in community structure (Brown et al., 2022).

The above studies indicated that fertilization affects community structure through some critical processes, including trait‐based functional response, reordering of species, niche dimensionality, and biomass (in)variability, which eventually represents specific trends in changes of SADs. Those processes have been investigated individually, although the integrative process has not been shown clearly. Therefore, regarding the effects of fertilization on the overall community structure, it should reveal (1) mechanisms that the species reordering driven by the plant responses causes characteristic trends in temporal changes in the shape and species composition of RAD, (2) reasons of that those trends are ubiquitous in various systems, and (3) conditions that species diversity does or does not associate with biomass increment. To understand the underlying processes in the effects of fertilization on the plant community, we need a theory that links fertilization (including improvement of productivity of plants) and community structure.

One candidate of such a theory (model) is the colonization model (or Levins model; Hastings, 1980; Levins, 1969; Levins & Culver, 1971), which was developed to understand the mechanisms driving species coexistence (Kinzig et al., 1999; Lehman, 2000; Tilman, 1994). The colonization model focuses on competition among propagules for spatially distinct areas to colonize and assumes that in an encounter between two species at a given local site, competitively inferior species gets eliminated from the site. This dynamic could promote coexistence under various trade‐offs in species properties, for example, competition–colonization and colonization–mortality trade‐offs, where a higher competitive ability is accompanied by a lower colonization rate and a lower survivorship, respectively. (Kinzig et al., 1999; Lehman, 2000; Tilman, 1994). Recently, Yamauchi, Ito, and Shibasaki (2021) reported that, under the competition–fecundity trade‐off (that is a modification of the competition–colonization trade‐off of the previous study), the colonization model can explain trends in species abundance, such as the shape of the RAD or the position of a given species within an RAD. This finding suggests that interspecific competition may be a critical factor in species diversity and community structure.

We assessed if the colonization model, including a competition–fecundity trade‐off, could explain trends observed in long‐term fertilization experiments, wherein fertilization is presumed to change plant fecundity. We also show that the trends can occur even without the drastic change in total biomass depending on conditions.

## 2 MODEL SPECIFICS

We considered an ecological community involving *n* potential species, in which each species was indexed as *i* = 1, 2, 3,…, *n*. The index coincided with competitive ability, where a larger *i* value corresponded to increasingly competitively inferior species. The habitat consisted of multiple sites that were habitable for all species; each site was either empty or colonized by a single species at each moment in time. Colonies continuously reproduced and dispersed propagules that colonized other sites. The probability that a propagule arrived at a certain site and developed to the colony growth stage was represented as *q*, also known as the basal colonization rate. We note that 1–*q* simultaneously includes the probabilities of both a failure to arrive and a failure to reach the colony growth stage. At an empty site, a propagule that successfully reached the colony growth stage eventually established a new colony at that site. However, when a site was already colonized, competition occurred between the site inhabitant and the colonizing intruder at the colony growth stage, wherein competition was assumed to follow “displacement competition,” that is, the competitively superior species immediately defeats the inferior species and replaces it in a site. We also considered that competitiveness is accompanied by cost. We assumed that competitiveness reduced the productivity of propagules, following the competition–fecundity trade‐off, which has been suggested in empirical studies. Ghalambor and Martin (2001) reported fecundity–survival trade‐offs in bird species. If the survivorship affects competition, it can be regarded as the fecundity–competition trade‐off. Rees et al. (2001) show a trade‐off in tree species between vertical growth in high‐light conditions and survivorship in low‐light conditions. If the vertical growth in the canopy and the offspring survivorship in the understory influence fecundity via the light gain and competitiveness via the ability of habitat occupation, respectively, this may also correspond to the fecundity–competition trade‐off. Therefore, the reproductive rate of *i*‐th species, *f*_*i*_, was assumed to increase with each increment of competitive inferiority. A site with an established colony would be returned to an empty state following disturbance, which occurred at a probability of *m*, also known as the extinction rate.

Continuous‐time dynamics in the frequency of sites occupied by *i*‐th species, *p*_*i*_, can be expressed by

(1) $$\frac{dp_{i}}{\mathrm{dt}}=qf_{i}p_{i}\left(1–\sum_{j=1}^{i}p_{j}\right)–q\sum_{j=1}^{i–1}f_{j}p_{j}p_{i}–mp_{i}$$

(Kinzig et al., 1999; Lehman, 2000; Tilman, 1994; Yamauchi et al., 2023; Yamauchi, Ito, & Shibasaki, 2021). Previous studies have made extensive analyses of equilibrium distributions of species frequencies (Kinzig et al., 1999; Lehman, 2000; Tilman, 1994; Yamauchi, Ito, & Shibasaki, 2021). By applying an approximation to continuous competitiveness, the equilibrium species distribution of Equation (1) can be analytically solved, wherein an infinite number of species can coexist with a smooth continuous abundance distribution. In addition, when competitiveness is discrete, with a finite number of species, the equilibrium species distribution can be numerically derived as

(2) $$p_{i}^{*}=\left\{\begin{array}{cc} 1–\sum_{j=1}^{i–1}p_{j}^{*}–\frac{1}{f_{i}}\left(\sum_{j=1}^{i–1}f_{j}p_{j}^{*}+\frac{m}{q}\right) & \text{if this is positive}\\ 0 & \text{otherwise} \end{array}\right..$$

Since the equilibrium frequency of a focal species *p*_i_* is described by its own fecundity *f*_*i*_ and the frequencies of more competitive species, that is, *f*_*j*_ *p*_j_* for ∀ *j* < *i*, the frequency of all species can be determined by applying a forward recursive procedure from *i* = 1 to *n*. In this procedure, species *i* first appears when the species achieves *p*_i_* > 0 in the absence of more competitive species (i.e., *p*_j_* = 0 for ∀ *j* < *i*), a condition expressed as

(3) $$f_{i}>\frac{m}{q},$$

from Equation (2).

The species frequency distribution tends to have a characteristic shape under discrete competitiveness, where some species are reduced to low frequencies or driven to extinction, resulting in a serrated pattern in the equilibrium species distribution when considered on the axis of competitive ability (Kinzig et al., 1999; Lehman, 2000; Tilman, 1994; Yamauchi et al., 2023; Yamauchi, Ito, & Shibasaki, 2021). We focused on discrete competitiveness for two reasons. First, results derived under continuous competitiveness do not reflect natural communities, which cannot in reality contain an infinite number of species. Second, Yamauchi, Ito, and Shibasaki (2021) indicated that empirical data were consistent with the community structures derived under discrete competitiveness.

We assumed that fecundity was an increasing function of competitive inferiority given the competition–fecundity trade‐off. Fecundity tends to saturate at high competitive inferiority because environmental factors limit maximum fecundity. The fecundity of an individual with an *i*‐th order of competitiveness was expressed as

(4) $$f_{i}=\alpha \frac{1–\mathrm{Exp}\left[–\mathrm{\beta i}/n\right]}{1+\mathrm{Exp}\left[–\mathrm{\beta i}/n\right]},$$

where *α* and *β* represent the maximum fecundity and magnitude of saturation, respectively. We considered that fertilization should improve the fecundity of all species by reducing environmental limitations, corresponding to increases in *α*. However, fecundity may also be limited by an organism's physiological capacity, which would be unaffected by relaxed environmental limits. Therefore, increased fecundity as a result of fertilization is most exaggerated in species with an initially low fecundity that are not limited by physiology. This may lead to high saturation in fecundity following fertilization, corresponding to increases in *β*.

**FIGURE 1:**
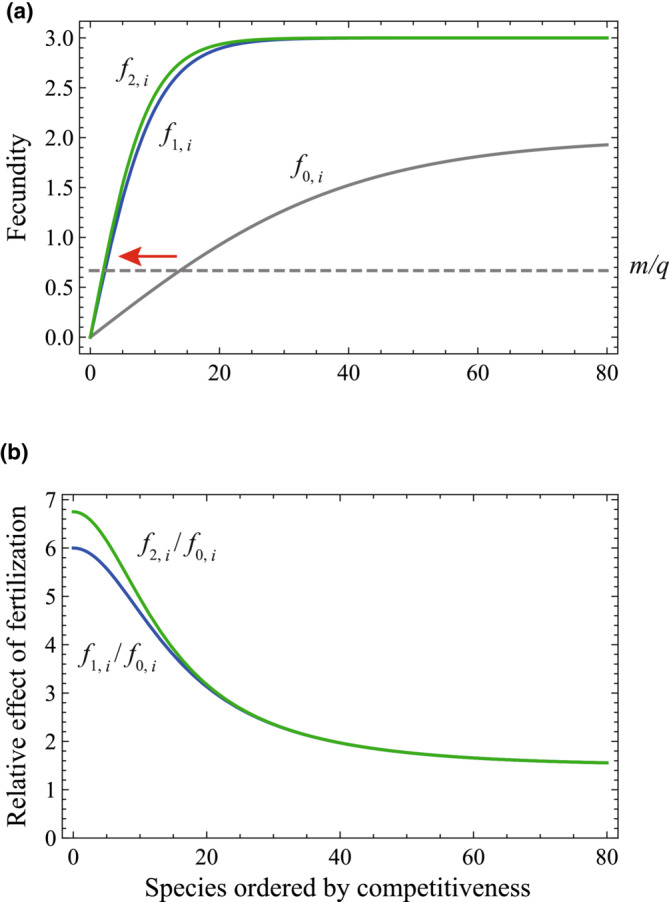
(a) Fecundity functions used in analyses representing competition–fecundity trade‐off. The original condition was expressed as *f*_0,*i*_ = 2(1−Exp[−4*i*/80])/(1 + Exp[−4*i*/80]), and fertilization was expressed as *f*_1,*i*_ = 3(1−Exp[−16*i*/80])/(1 + Exp[−16*i*/80]) or *f*_2,*i*_ = 3(1−Exp[−18*i*/80])/(1 + Exp[−18*i*/80]). (b) Relative improvements in fecundities following fertilization. It was assumed that 80 species could exist within the simulation.

We consider that long‐term fertilization improves species productivity, which eventually increases species fecundities. La Pierre and Smith (2015) investigated responses of functional trait expressions of grassland species in fertilization experiment, which showed that one response trait (community aggregate height) strongly correlated with variation in aboveground net primary productivity with long‐term nutrient additions. This may justify our assumption that the plant response to fertilization raises the fecundity via improving productivity. In the present analysis, we consider changes in the competition–fecundity trade‐off as illustrated in Figure 1a. First, *f*_0,*i*_ was assumed to be the original fecundity function with *α* = *α*_0_ = 2 and *β* = *β*_0_ = 4, and then, *f*_1,*i*_ and *f*_2,*i*_ represented two fertilization scenarios with *α* = 3 (=1.5*α*_0_) and *β* = 16 (=4*β*_0_), and *α* = 3 (=1.5*α*_0_) and *β* = 18 (=4.5*β*_0_), respectively. Figure 1b shows the relationship between relative improvements in fecundity under fertilization, where improvements are more pronounced in highly competitive species, consistent with an increase in *β*.

**FIGURE 2:**
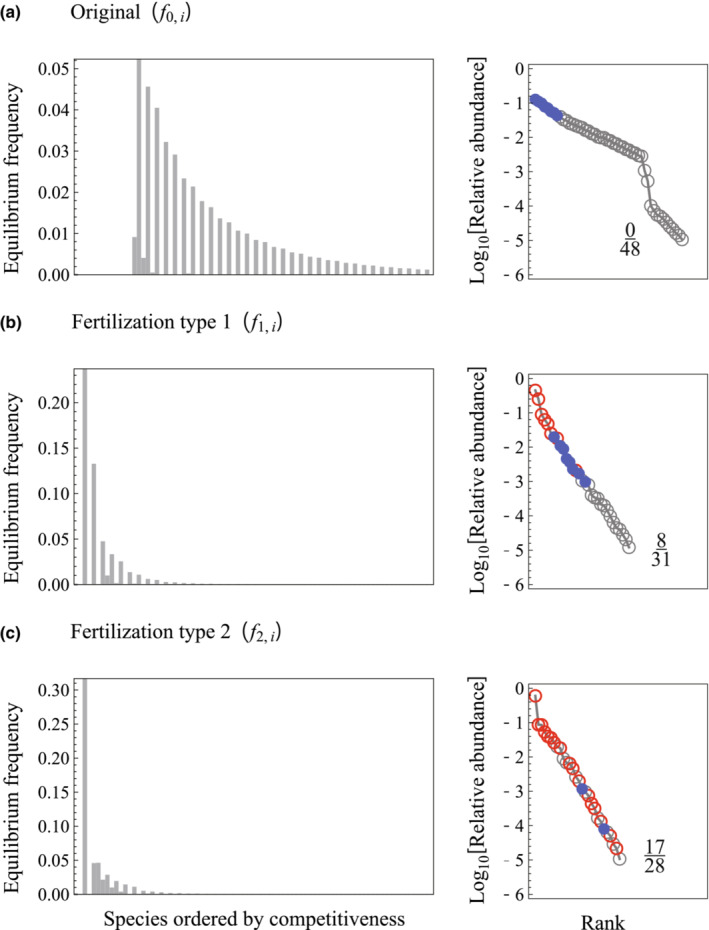
Equilibrium frequency distributions of sites with each species and RADs under fecundities illustrated in Figure 1. We excluded species with a relative abundance <10^−5^ from the RADs. Blue points represent the eight most abundant species in Figure 1a, and red circles represent species that were absent in Figure 1a. Fraction numbers on the RADs represent species compositions, where the denominator and numerator indicate the total number of species and that of lost species by fertilization, respectively. Parameters were *n* = 80, *q* = 0.3, and *m* = 0.2.

The equilibrium frequency distributions of sites occupied by species are provided in Figure 2. As described previously, the distributions showed serrated patterns, where high and low frequencies appeared in an alternating fashion along the axis of competitiveness. These frequency distributions can be transformed to an RAD (Figure 2, note that species with a relative abundance <10^−5^ were excluded from the RAD). The difference in equilibrial RADs between pre‐ and postfertilization indicated that fertilization drove changes in species composition. In the sense of a “labeled SAD” (McGill et al., 2007), we distinguished some species in the RADs based on their abundance status (Figure 2). The two fertilization functions, *f*_1,*i*_ and *f*_2,*i*_, were similar to each other in functional form (see Figure 1), but the magnitude of change in species composition following fertilization was notably different (see Figure 2). Fertilization under *f*_1,*i*_ resulted in the appearance of only seven new species, whereas fertilization with *f*_2,*i*_ led to the appearance of 22. Despite the difference, fertilization in both cases led to a decline in the total number of species, shifted the composition of the most abundant species, and resulted in an RAD with a steeper slope.

**FIGURE 3:**
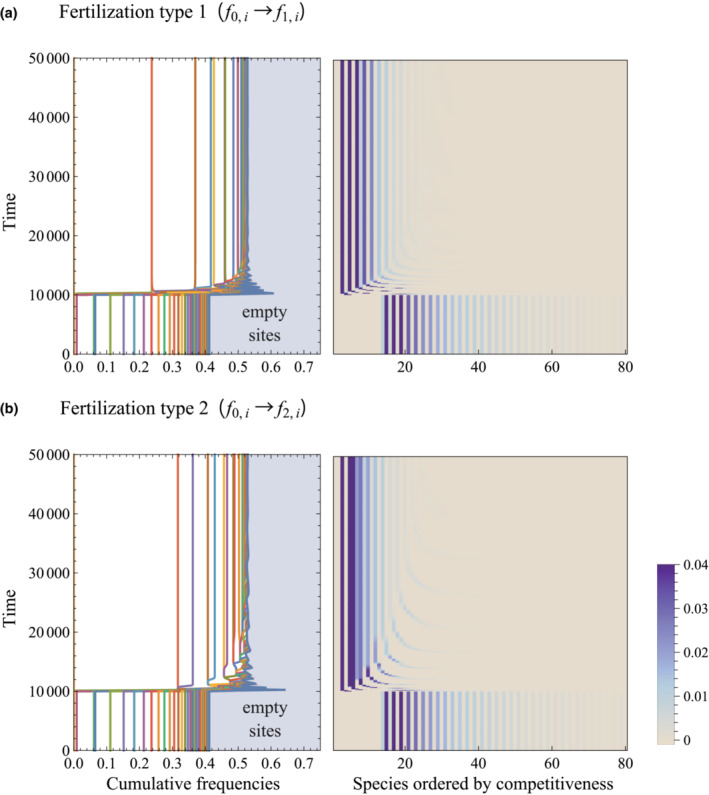
Dynamic time series of frequencies of sites with each species. Fertilization was initiated at *t* = 10,000. Left panels show cumulative frequency plots, where a space between two curves represents a specific frequency. Species are arranged by competitive ability from left to right. Right panels represent density plots of the frequencies of sites with each species on competitive ability. An immigration term was included at a rate of 10^−10^. Parameters were *n* = 80, *q* = 0.3, and *m* = 0.2.

**FIGURE 4:**
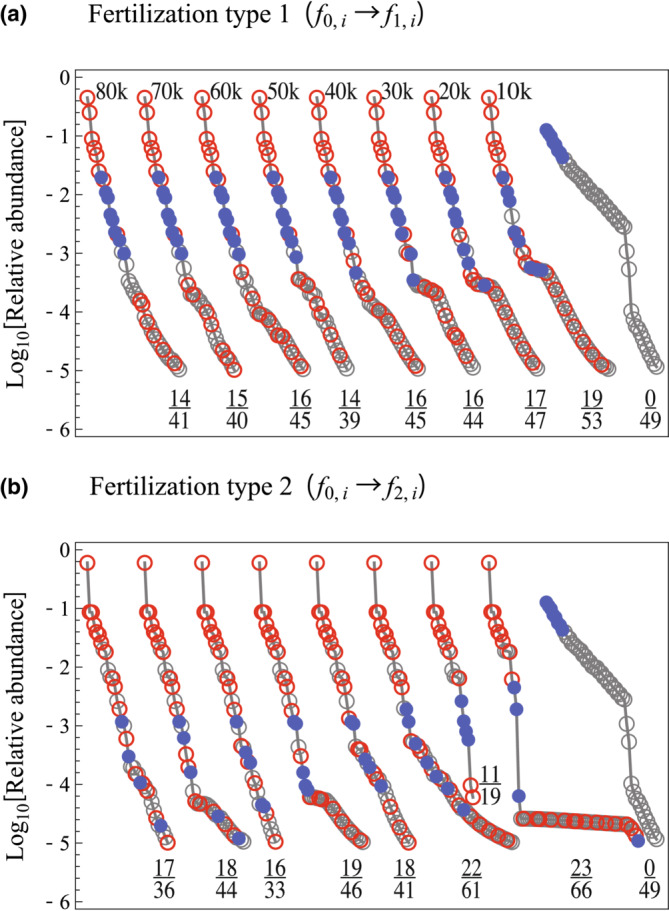
Temporal change in an RAD following fertilization, in which k means 1,000 timesteps after the initiation of fertilization. Fraction values on each plot represent species composition, where the denominator and numerator indicate the total number of species and that of lost species by fertilization, respectively. Parameters were *n* = 80, *q* = 0.3 and *m* = 0.2.

We further investigated transitory processes between equilibria before and following fertilization. Figure 3 illustrates the frequency dynamics of sites where fertilization was initiated at *t* = 10,000, after which a transition to another equilibrium began. In these simulations, continuous immigrations were introduced to Equation (1) at a rate of 10^−10^, to avoid complete extinction and enable a dynamic response of all species to fertilization treatments. Generally, communities gradually converged to a new equilibrium following fertilization. In this case, the fertilization reduces frequencies of empty sites, which is consistent with an increment of the community biomass. Transitory RADs are shown in Figure 4, illustrating temporal change in community structure. In contrast to Figure 2, community composition was unlikely to reach equilibrium even after *t* = 80,000. Generally, as shown in Figure 2, new species rapidly appeared after fertilization was initiated, but the steepening of the RAD slope progressed gradually. The number of species in a community increased during transitions, likely a result of a large number of species with low relative abundance. Fertilization drives the emergence and gradual extinction of species, where they can exist simultaneously at the transient stage, resulting in the temporal increment of the species richness. Corresponding trends were observed in fertilization experiments in peat fields (Kirkham et al., 1996), although we consider that its occurrence may depend on the turnover rate of the community. As these low‐abundance species were later excluded from the community, total richness tended to gradually decrease in the approach to equilibrium.

**FIGURE 5:**
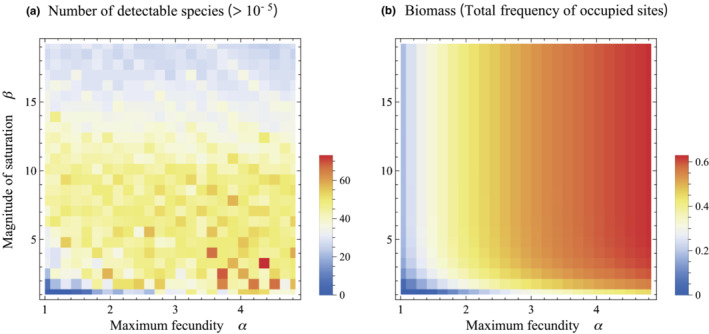
Species richness (a) and biomass (b) at equilibrium under competition–fecundity trade‐offs with varying combinations of intensity of saturation, *α*, and maximum fecundity, *β*. Parameters were *n* = 80, *q* = 0.3, and *m* = 0.2.

Here, a colonization model with a competition–fecundity trade‐off well‐represented three trends typical to fertilization experiments: declines in species richness, the replacement of initially abundant species by those that were initially rare or absent, and a steeper RAD slope. Fertilization had two attributes, fecundity increment and saturation. We also investigated the relative contributions of these two factors to the shape of the RAD curve. Figures S1–S3 show that strong saturation (increased *β*) had a greater effect on the shape of the RAD than did the fecundity increment (increasing *α*). To investigate the effects of *α* and *β* on the species richness comprehensively, we plot the number of detectable species (frequency higher than 10^–5^) at equilibrium with a varying set of *α* and *β* in Figure 5a. The figure indicates significant effects of saturation of the trade‐off function (large *β*) on the decline of species richness.

This suggests three possible mechanisms. First, strong saturation of fecundity at low competitiveness may decrease species richness via the exclusion of less competitive species, given their loss of fecundity advantage. Second, the appearance of new species may be the result of shifts in trade‐offs reflecting *m* and *q*. Given that *f*_*i*_ > *m*/*q* is a necessary condition for the persistence of *i*‐th species (Equation 2), the leftward shift of the trade‐off curve for *m*/*q* could enable the persistence of more competitive (but less fecund) species. Finally, the steepening of the slope of the RAD is a result of the strong saturation in fecundity (*f*_1,*i*_ and *f*_2,*i*_ in Figure 1). When saturation is weak, the serrated pattern of the frequency distribution tends to result in two distinct phases in the RAD, with abundant and less abundant species that have close competitiveness values (Figure 2). Under strong saturation, the frequency distribution rapidly declines with decreasing competitiveness. This steepens the first phase of the RAD, consisting of abundant species, leading to a continuous shape. Combined with declines in species richness, an RAD with a continuous steep slope is produced (Figure 2).

Figure S3b also represents a significant implication of the effect of fertilization. In the figure, the total biomass (i.e., frequency of empty sites) does not change notably, although fertilization significantly modifies the structure and shape of RAD (Figure S2c). To explore the effects of the shape of trade‐off function on the biomass, we plot the total frequency of occupied site at equilibrium with a varying set of *α* and *β* in Figure 5b. The figure indicates that the saturation of the trade‐off function (large *β*) is unlikely to influence the community biomass. According to Figure 5a,b, fertilization can alter the community structure with remaining similar biomass under the intensifying saturation without the overall increment of fecundity in competition–fecundity trade‐off (Harpole et al., 2016). This result indicates that biomass increment is not a crucial factor in the influence of fertilization on the structure of the plant community.

In our model, similar fertilization treatments led to substantially different outcomes in community composition. The scenario with fecundity function *f*_1,*i*_, led to the appearance of eight new species (out of 31), only at the highest‐ranked positions (Figure 2b). The similar function *f*_2,*i*_ led to the appearance of 17 new species (out of 28) at multiple positions on the RAD (Figure 2c). We note that species with odd competitive ranks tended to achieve high abundances in Figure 2a,b, but those with even ranks were most abundant in Figure 2c. Therefore, differences in the attributes of the dominant species (Figure 2a vs. c) drove species replacement after fertilization, with greater emergence of new species in communities in the latter.

In the above analysis, we considered the smooth trade‐off functions, where the fecundity monotonically increases with the increasing competitive inferiority. To test the robustness of our results to this assumption, we also check the effects of fluctuation of trade‐off functions on the changes of RADs under fertilization. Figure S4 adopts trade‐off functions that randomly fluctuate *f*_0,*i*_ and *f*_1,*i*_ in Figure 1 with ±5% for each species (Figure S4a). Figure S4b illustrates that the fluctuation of the trade‐off function tends to decline the overall species number notably. However, qualitative tendencies in the fertilization event are likely to reserve, in which both diversity and evenness in the community decrease after fertilization.

## 3 DISCUSSION

Following Kempton's (1979) report that fertilization altered species composition and the shape of the RAD, many similar reports have emerged based on different systems (Avolio et al., 2019; Brown et al., 2022; Collins et al., 2008; Kirkham et al., 1996; Sand‐Jensen et al., 2008). Trait‐based research revealed that plant responses to fertilization were essential for the changes in community structure (Avolio et al., 2019; Brown & Zinnert, 2021; Dickson & Gross, 2013; La Pierre & Smith, 2015; Suding et al., 2005). However, some questions have remained yet. How does the species reordering driven by the plant responses cause characteristic trends in temporal changes of the shape and species composition of RAD? Why are those trends ubiquitous in various systems? We show that interspecific competition may be a critical force driving change in community structure in response to fertilization. This in turn suggests that interspecific competition may be a key determinant of species diversity. Our colonization model, which included a competition–fecundity trade‐off in the manner of Yamauchi, Ito, et al. (2021), explained empirical trends observed from fertilization studies, further highlighting the importance of interspecific competition.

DeMalach et al. (2017) and DeMalach and Kadmon (2017) indicated that light competition determined the structure of plant communities. They stated that nutrient enrichment increased the asymmetry of light partitioning among tall and short plants, which resulted in the decline of species richness. If some conditions are satisfied, their scenario may correspond with our results. First, fertilization did not alter the order of competition rank. Second, the increment of competitive asymmetry was accompanied by the increment of fecundity, which may occur in competitively superior species, that is, tall species. It is expected that more detailed discussions may be possible if we have information about fecundity.

It should be noticed that fertilization can result in various influences to plant communities via multiple steps. Previous studies suggested that fertilization of nutrient(s) causes responses of plants in trait expression (La Pierre & Smith, 2015; Suding et al., 2005), which drives reordering of species abundance, resulting in changes in the community structure (Avolio et al., 2019). The present analysis considers that the functional responses of plant traits to fertilization increase the fecundity of each species. The analysis shows that such a fecundity improvement can modify a interaction scheme in the community, which results in the appearance of more competitive species and the reordering of species ranks in RADs under the hierarchical interspecific competition.

It was reported that the addition of multiple types of nutrients tends to intensify the effects of fertilization, including the significant decline of diversity (Avolio et al., 2019; Harpole & Tilman, 2007). We expect that the addition of multiple nutrients improves plant productivities notably, which alters the competition–fecundity trade‐off, resulting in the remarkable change in the community structure. On the contrary, Harpole et al. (2016) reported that in some cases, species diversity can decline without biomass increment typically under additions of multiple nutrients. Nevertheless, our analysis shows that community structure can change either with or without significant increment of biomass, depending on the shape of the trade‐off function (see Figure 5). When the addition of multiple nutrients causes a notable saturation of trade‐off function without the drastic increment of fecundity, the number of species can decrease with keeping a similar level of community biomass. Such situations may be possible if physiological constraint strongly restricts the fecundity. This might be consistent with some cases in Harpole et al. (2016), where the diversity declines without biomass increment. More research with a specific focus on the relationship between productivity, fecundity, and nutrient number should be addressed in future research to try and field‐test the validity of model results.

Furthermore, fertilization may also indirectly affect community structure. For example, long‐term fertilization may modify soil properties and microbial communities (Wang et al., 2019; Wen et al., 2020). These factors may then influence plant community structure. However, if these interactions and effects can be incorporated eventually into species fecundity (i.e., trade‐off function), our approach will remain valid. Similarly, even if fertilization led to unexpected or alternative effects, our predictions would remain robust provided the effects involved change in fecundity. However, we note that community responses would be slowed if effects on fecundity included a time lag.

We demonstrated that small differences in fecundity can lead to substantially different community outcomes, which may have implications beyond fertilization. For example, regional differences in community composition may be the result of small differences in fecundity. It was also reported that the addition of nitrogen and phosphorus together increased variability of plant community across space (Koerner et al., 2016). Our analyses suggest that the wide heterogeneity observed in community structure may be the result of small differences in fecundity. In addition, this suggests that small environmental changes may cause drastic changes in community composition. The present analysis suggests that we should reinvestigate a relationship between fertilization, plant fecundities, and interspecific competition to understand determinant processes of community structure. The presented theory will be tested by empirically studying the competition–fecundity trade‐off in communities.

## AUTHOR CONTRIBUTIONS

**Atsushi Yamauchi:** Conceptualization (lead); formal analysis (lead); funding acquisition (lead); investigation (lead); software (lead); validation (equal); visualization (lead); writing – original draft (lead); writing – review and editing (equal). **Koichi Ito:** Validation (equal); writing – review and editing (equal). **Shota Shibasaki:** Validation (equal); writing – review and editing (equal).

## CONFLICT OF INTEREST STATEMENT

The authors declare that they have no conflict of interest.

## Supporting information


Data S1:

## Data Availability

Mathematica notebooks are uploaded to Zenodo (10.5281/zenodo.6500386).
