# Supplementary material for: Competition model explains trends of long‐term fertilization in plant communities
Source: Ecol Evol. 2023 Feb 14;13(2):e9832. doi: 10.1002/ece3.9832 (PMC9929124; doi:10.1002/ece3.9832)
Supplement: Supplementary file 1 — Data S1: [file ECE3-13-e9832-s001.zip › ECE3_9832_Supplementary.pdf]

CAPTIONS OF SUPPLEMENTARY FIGURES

Supplementary Figure 1. (a) Fecundity functions used in analysis representing competition-fecundity trade-off. The original condition was assumed as $f_{0,i}$, and fertilization was expressed as $f_{3,i}$ = 3.5(1-Exp[-4i/80])/(1+Exp[-4i/80]) (increasing fecundity only, $\alpha = 1.5\alpha_0$ and $\beta = \beta_0$) or $f_{4,i}$ = 2(1-Exp[-15i/80])/(1+Exp[-15i/80]) (intensifying saturation only, $\alpha = \alpha_0$ and $\beta = 3.75\beta_0$). (b) Relative improvements in fecundities following fertilization. It was assumed that 80 species could exist within the simulation.

Supplementary Figure 2. Equilibrium frequency distributions of sites with each species and RADs under fecundities illustrated in Supplementary Figure 1. RADs excluded species with a relative abundance < 10^{-5}. Blue points represent the 8 most abundant species in S1a and red circles represent species that were absent in S1a. Fraction values on each plot represent species composition, where the denominator and numerator indicate the total number of species and that of lost species by fertilization, respectively. Parameters were $n = 80$, $q = 0.3$ and $m = 0.2$.

Supplementary Figure 3. Dynamic time series of frequencies of sites with each species. Fertilization was initiated at t = 10,000. Left panels show cumulative frequency plots, where a space between two curves represents a specific frequency. Species are arranged by competitive ability from left to right. Right panels represent density plots of the frequencies of sites with each species on competitive ability. An immigration term was included at a rate of 10^{-10}. Parameters were $n = 80$, $q = 0.3$ and $m = 0.2$.

Supplementary Figure 4. Effects of randomness in trade-off on the transition of RAD. (a) Fecundity functions representing competition-fecundity trade-offs, in which $\tilde{f}_{0,i}$ and $\tilde{f}_{1,i}$ correspond with $f_{0,i}$ and $f_{1,i}$ in Fig.1, respectively, with ±5% of even randomness in the fecundity. (b) Temporal change in an RAD following fertilization. Parameters and conditions are identical to those

in Fig.4.

Supplementary Figure 1

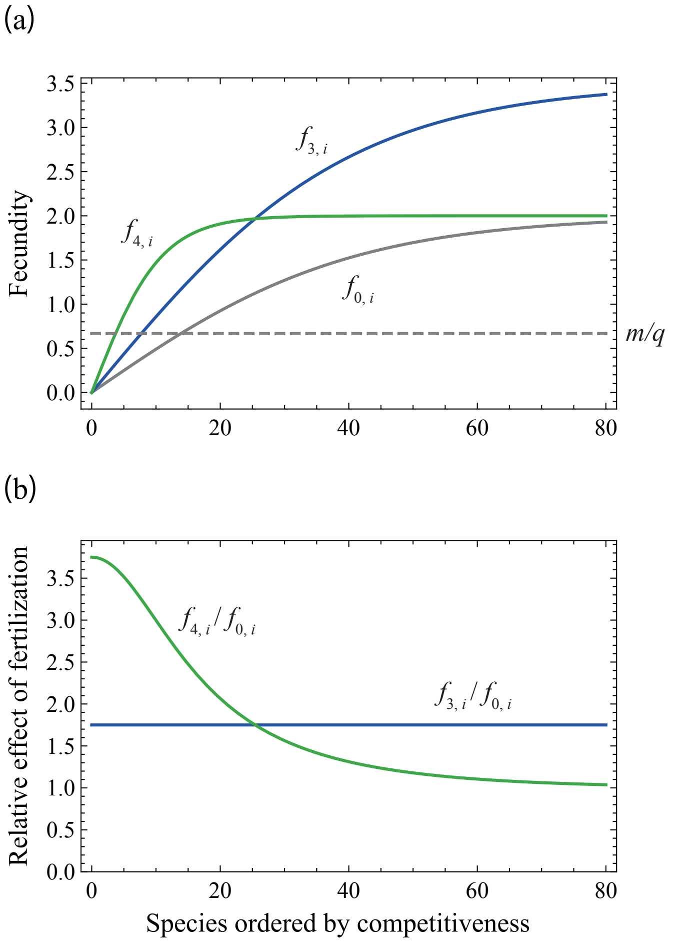

Supplementary Figure 2

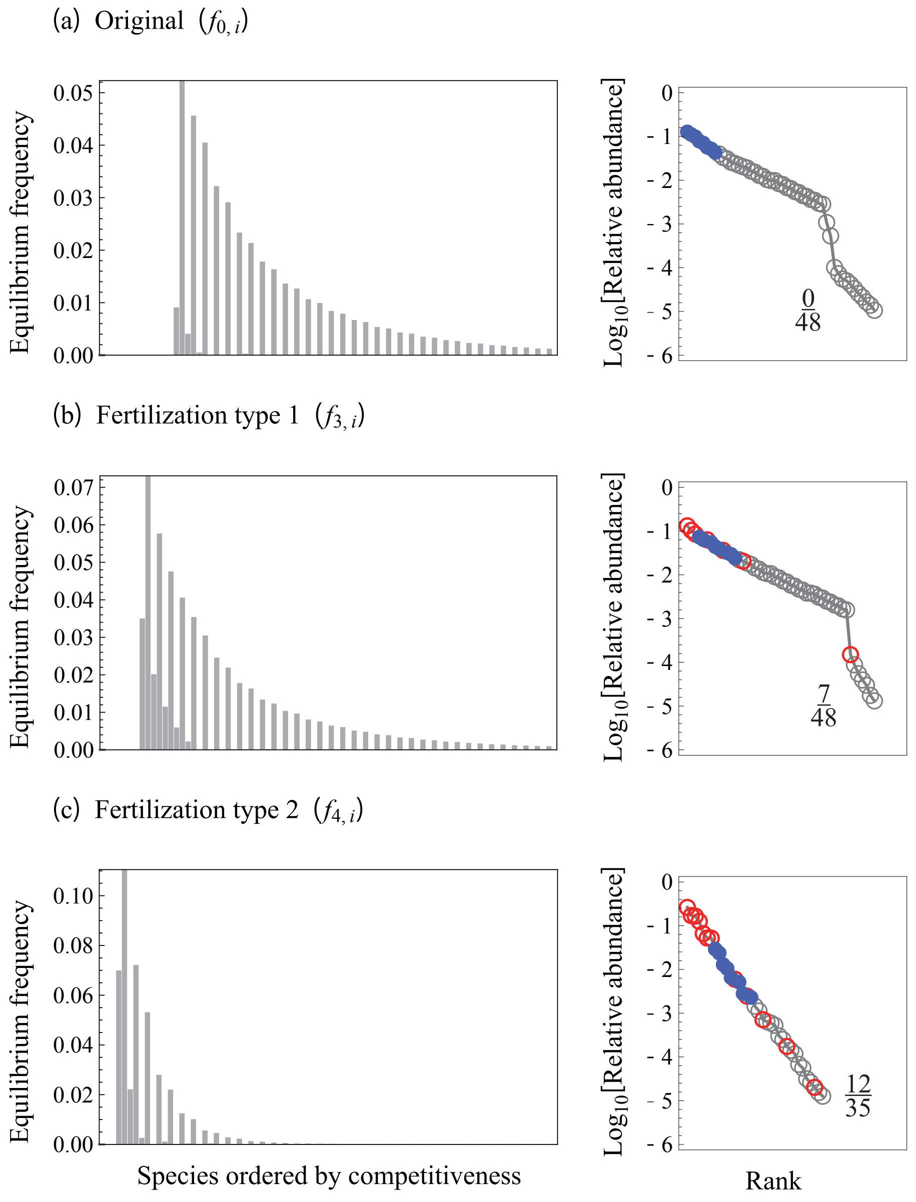

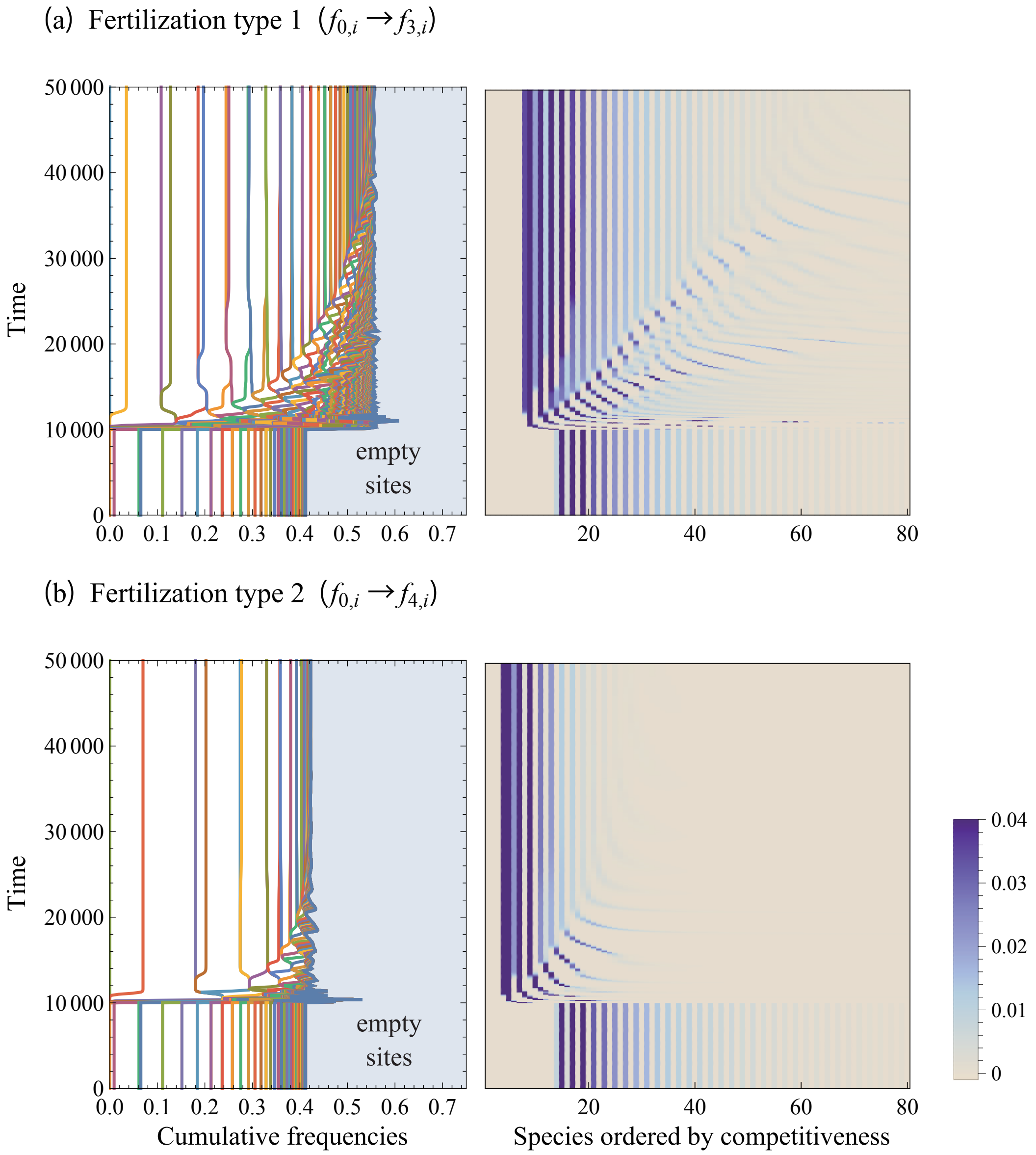

(a) Fertilization type 1 $(f_{0,i} \rightarrow f_{3,i})$
(b) Fertilization type 2 $(f_{0,i} \rightarrow f_{4,i})$
Time
50 000
40 000
30 000
20 000
10 000
0
empty
sites
0.0 0.1 0.2 0.3 0.4 0.5 0.6 0.7
20 40 60 80
Cumulative frequencies
Species ordered by competitiveness
0.04
0.03
0.02
0.01
0

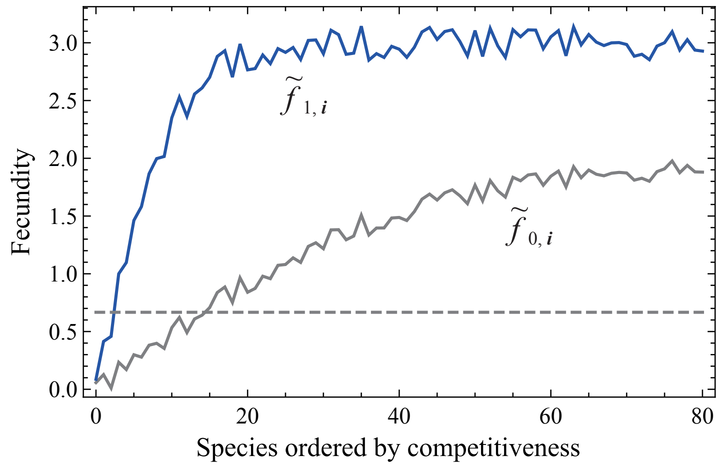
Fecundity
Species ordered by competitiveness
$\tilde{f}_{1,i}$
$\tilde{f}_{0,i}$
0.0
0.5
1.0
1.5
2.0
2.5
3.0
0
20
40
60
80

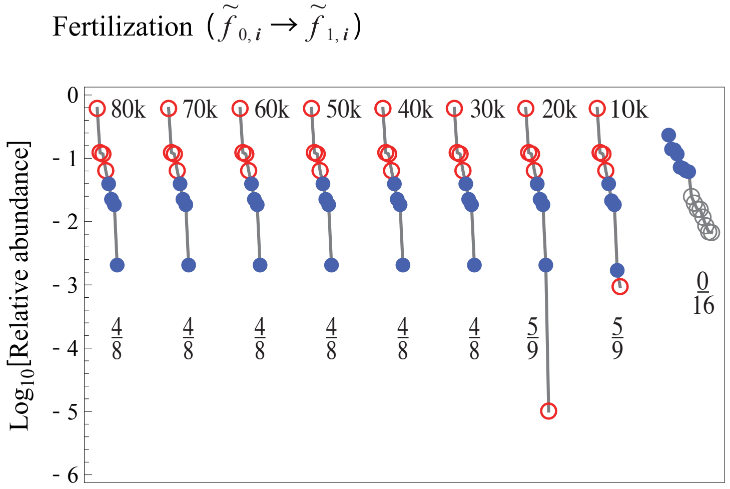
Fertilization $(\tilde{f}_{0,i} \to \tilde{f}_{1,i})$
Log10[Relative abundance]
80k
70k
60k
50k
40k
30k
20k
10k
4/8
4/8
4/8
4/8
4/8
4/8
5/9
5/9
0/16
0
- 1
- 2
- 3
- 4
- 5
- 6
